# Laboratory Surveillance of Bovine Brucellosis: Predictors of Rose Bengal Test Positivity in Mpumalanga Province, South Africa (2021–2024)

**DOI:** 10.3390/vetsci13030284

**Published:** 2026-03-18

**Authors:** Themba Titus Sigudu, Phoka Caiphus Rathebe, Masilu D. Masekameni, Tintswalo Mercy Hlungwani, Khuthatshelo Vincent Mphaga, James Wabwire Oguttu

**Affiliations:** 1 Division of Health and Society, School of Public Health, Faculty of Health Sciences, University of the Witwatersrand, Johannesburg 2000, Gauteng, South Africa; tintswalo.hlungwani@wits.ac.za; 2 Department of Environmental Health, Faculty of Health Sciences, Doornfontein Campus, University of Johannesburg, Johannesburg 2000, Gauteng, South Africa; prathebe@uj.ac.za (P.C.R.); mphagakv@gmail.com (K.V.M.); 3 Developmental Studies, School of Social Sciences, University of South Africa, Pretoria 0003, Gauteng, South Africa; maseksd@unisa.ac.za; 4 Department of Agriculture and Animal Health, College of Agriculture and Environmental Sciences, University of South Africa, Johannesburg 1709, Gauteng, South Africa; joguttu@unisa.ac.za

**Keywords:** epidemiology, seasonal variation, spatial heterogeneity, temporal trends, zoonotic disease

## Abstract

Bovine brucellosis is an endemic zoonotic disease in South Africa with significant consequences for livestock productivity and public health. Although routine laboratory surveillance data from the Rose Bengal Test (RBT) are widely collected, they are seldom used to investigate temporal and spatial patterns of disease detection. This study aimed to examine temporal, seasonal, and spatial predictors of RBT positivity for bovine brucellosis in Mpumalanga Province, South Africa. A retrospective observational study was conducted using routine laboratory records from the Mpumalanga Provincial Veterinary Laboratory between January 2021 and December 2024. The dataset included all bovine serum samples with complete information on testing date, municipality, and RBT results. Laboratory submissions were recorded as batches, defined as groups of serum samples submitted together to the laboratory as part of a single surveillance or investigation event. The primary outcome was batch-level RBT positivity, defined as the presence of at least one RBT-positive serum sample within a submission batch. Temporal (year of testing), seasonal (season of submission), and spatial (local municipality area) variables were evaluated as predictors of RBT positivity using logistic regression models. Mixed-effects logistic regression accounted for the clustering of submissions within municipalities. A total of 568 submission batches comprising 67,974 serum samples were analysed, of which 6182 tested positive, yielding an overall positivity of 9.1%. RBT positivity increased significantly in 2023 compared with 2021 (AOR = 2.47; 95% CI: 2.27–2.68). Seasonal variation was observed, with higher odds of positivity in spring (AOR = 1.80; 95% CI: 1.65–1.97) and lower odds in autumn and winter relative to summer. Mixed-effects modelling indicated significant residual spatial heterogeneity in RBT positivity across municipalities. Routine laboratory surveillance data can provide valuable epidemiological insights into the temporal, seasonal, and spatial dynamics of bovine brucellosis detection and support risk-based surveillance strategies in endemic livestock systems.

## Simple Summary

Bovine brucellosis is a common animal disease in South Africa that affects cattle health, reduces productivity, and poses significant risks to human health. This study used routine laboratory test records from Mpumalanga Province of South Africa to examine when and where positive cases of bovine brucellosis were most likely to occur. Data from 2021 to 2024 showed that about 9% of the tested cattle samples were positive. The likelihood of positive results increased over time, was highest during spring, and was greater in municipalities classified as high risk. These findings show that routinely collected laboratory data can be used to identify high-risk periods and areas, supporting more targeted and effective brucellosis surveillance and control programmes.

## 1. Introduction

Bovine brucellosis remains one of the most important endemic zoonotic diseases affecting livestock production and public health globally, particularly in low- and middle-income countries where control and eradication efforts are constrained by resource limitations and complex production systems [1,2]. Caused by Brucella abortus in cattle, the disease is associated with reproductive losses, including abortion, infertility, and reduced milk production, leading to substantial economic impacts at farm and national levels [3]. In addition, bovine brucellosis poses a significant zoonotic risk, especially for individuals involved in livestock handling, veterinary services, and abattoir work [4].

Beyond its epidemiological importance, brucellosis is characterized by complex host–pathogen interactions, with immune dysregulation playing a central role in disease persistence and clinical expression. Experimental and field studies have demonstrated that Brucella abortus infection is associated with altered expression of cytokine-related genes, reflecting the pathogen’s ability to modulate both innate and adaptive immune responses [5]. Further evidence indicates that cytokine signalling in brucellosis follows distinct biological rhythms, influencing inflammatory responses and disease progression at the host–pathogen interface [6].

In South Africa, bovine brucellosis is a controlled disease under the Animal Diseases Act, with surveillance and control relying on a combination of routine testing, movement control, and vaccination programmes [7,8]. Despite these measures, the disease remains endemic in several provinces, including Mpumalanga, where both commercial and communal cattle production systems coexist [9,10]. The coexistence of these systems, together with extensive animal movement through auctions and abattoirs, complicates disease control and contributes to spatial and temporal heterogeneity in brucellosis occurrence [11].

Laboratory-based surveillance plays a central role in brucellosis monitoring, with the RBT widely used as a primary screening assay due to its low cost, simplicity, and high sensitivity under field conditions [12,13]. Routine diagnostic laboratory data generated through RBT testing offer a valuable but underutilized resource for understanding disease patterns, particularly when analysed longitudinally and spatially [14]. Recent studies have demonstrated that retrospective analyses of laboratory surveillance data can provide important insights into temporal trends, seasonal dynamics, and geographic clustering of brucellosis risk, thereby informing more targeted and efficient surveillance strategies [3,15].

Seasonal variation in brucellosis detection has been increasingly reported, with higher seropositivity often observed during warmer months, potentially reflecting seasonal calving patterns, increased animal movement, and heightened transmission risk [10,16]. Similarly, spatial heterogeneity at sub-provincial levels has been shown to persist even after accounting for temporal factors, underscoring the need for geographically stratified risk assessment approaches [15].

Despite the availability of routine laboratory data, few studies in South Africa have systematically examined predictors of RBT positivity using multivariable and hierarchical modelling approaches that account for temporal, seasonal, and spatial clustering simultaneously [17]. In Mpumalanga Province in particular, evidence remains limited on how RBT positivity varies across years, seasons, and local municipality areas, and how these patterns can be leveraged to support risk-based surveillance.

This study, therefore, aimed to investigate predictors of RBT positivity for bovine brucellosis in Mpumalanga Province, South Africa, using routine laboratory surveillance data collected between 2021 and 2024. By integrating temporal, seasonal, and spatial factors within a multivariable and mixed-effects modelling framework, the study seeks to characterize heterogeneity in RBT positivity and generate evidence to inform targeted surveillance, spatial prioritization, and improved brucellosis control strategies.

## 2. Materials and Methods

This study utilized secondary data that were fully anonymized and obtained from routine diagnostic records submitted to the Mpumalanga Provincial Veterinary Laboratory as part of provincial veterinary surveillance activities. All datasets were de-identified prior to analysis to protect confidentiality, and the study did not involve direct interaction with animals or human participants. Ethical approval was granted by the University of South Africa (UNISA), College of Agriculture and Environmental Sciences, Animal Research Ethics Committee (AREC-100818-024), and formal permission to access and use the data was obtained from the Department of Agriculture, Rural Development, Land and Environmental Affairs (DARDLEA). Given that the analysis relied solely on anonymized routine surveillance data, no additional animal ethics approval or owner consent was required.

### 2.1. Study Period and Location

The study was conducted in Mpumalanga Province, South Africa, using routine laboratory surveillance data collected over a four-year period from January 2021 to December 2024. Mpumalanga Province is located in the northeastern region of South Africa and is recognized as an important livestock-producing province characterized by a mix of commercial and communal cattle production systems. The province extends across diverse agro-ecological zones, ranging from highveld grasslands to subtropical lowveld areas, which influence cattle management practices, herd structure, and patterns of animal movement. These ecological and production system differences have implications for the transmission dynamics and detection of bovine brucellosis across the province.

Administratively, Mpumalanga is divided into 17 Local Municipality Areas (LMAs), which served as the primary spatial units of analysis. These municipalities differ in cattle population density, livestock trade intensity, proximity to abattoirs, and access to veterinary services, resulting in variations in surveillance intensity and testing volumes.

All serum samples analysed in this study were submitted to the MPVL through routine surveillance activities, including passive submissions, targeted control programmes, and outbreak investigations, thereby providing real-world data for evaluating temporal, seasonal, and spatial patterns in bovine brucellosis detection.

### 2.2. Study Design and Data Sources

This study employed a retrospective observational design based on routinely collected laboratory surveillance data to investigate predictors of RBT positivity for bovine brucellosis in Mpumalanga Province, South Africa. The analysis covered a four-year period, allowing assessment of temporal, seasonal, and spatial patterns in disease detection at the population level. Data were obtained from the MPVL, the primary diagnostic facility responsible for bovine brucellosis testing within the province.

The dataset comprised all bovine serum samples submitted for routine brucellosis screening during the study period. Laboratory submissions originated from passive surveillance activities, targeted control programmes, and outbreak investigations conducted across the province. Each laboratory record represented a batch submission and included information on the date of receipt, local municipality area, number of serum samples tested, and the number of RBT-positive samples.

Only records with complete information on testing date, local municipality area, and RBT results were included in the analysis. The use of routine diagnostic data provided a cost-effective and real-world surveillance dataset suitable for examining epidemiological patterns and identifying predictors of RBT positivity in an endemic livestock setting.

### 2.3. Data Management

All laboratory data were obtained in electronic format from the MPVL and were subjected to systematic data management procedures prior to analysis. The dataset consisted of batch-level records containing the date of receipt, local municipality area, number of serum samples tested, and number of RBT-positive samples. Data cleaning involved verification of variable completeness, removal of duplicate entries, and exclusion of records with missing or inconsistent information on testing date, municipality, or RBT results.

Date variables were converted and categorized to derive temporal indicators, including the year of testing and season of submission.

Seasons were defined as summer (December–February), autumn (March–May), winter (June–August), and spring (September–November).

Spatial identifiers were harmonized at the LMA level to ensure consistency across records. LMAs were subsequently grouped into collapsed spatial risk categories (low, moderate, and high risk) based on descriptive statistics, regression effect sizes, and spatial clustering analyses conducted during preliminary exploration.

All data were anonymized prior to analysis, with no farm-level or owner-identifying information included in the analytical dataset. The cleaned dataset was then imported into Stata version 18 for statistical analysis, with consistent variable coding and documentation maintained to ensure reproducibility and transparency.

### 2.4. Statistical Analysis

Descriptive statistics were used to summarize annual and seasonal testing volumes, numbers of RBT-positive samples, and overall RBT positivity, with corresponding 95% confidence intervals (CIs). Spatial distributions of RBT positivity were also examined across LMAs and collapsed spatial risk categories.

Bivariate associations between each explanatory variable and RBT positivity were assessed using unadjusted logistic regression models. Crude odds ratios (ORs), 95% CIs, and *p*-values were calculated to evaluate the strength of associations between temporal (year), seasonal, and spatial factors and RBT positivity. All explanatory variables considered epidemiologically relevant to the study objectives, including year of testing, season of submission, and local municipality area, were included in the multivariable model to control for potential confounding.

The outcome variable was batch-level RBT positivity, defined as the presence of one or more Rose Bengal Test-positive serum samples within a laboratory submission batch. Because laboratory submissions were nested within local municipality areas, a multilevel mixed-effects logistic regression model was fitted to estimate the association between temporal (year), seasonal (season of submission), and spatial (local municipality area) predictors and the likelihood of batch-level RBT positivity. The local municipality area was included as a random intercept to account for the clustering of submissions within municipalities.

Multivariable logistic regression was then performed to identify independent predictors of RBT positivity. Adjusted odds ratios (AORs) with 95% CIs were estimated, simultaneously adjusting for year, season, and collapsed LMA risk category. Model findings were interpreted in terms of both statistical significance and epidemiological plausibility. To account for the hierarchical structure of the data and potential clustering of serum samples within municipalities, mixed-effects logistic regression models were also fitted, incorporating LMA as a random intercept. This approach allowed assessment of residual spatial heterogeneity beyond the fixed temporal and seasonal effects. All statistical analyses were conducted using Stata version 18, and statistical significance was defined at a two-sided *p*-value of <0.05.

## 3. Results

### 3.1. Descriptive Statistics

#### 3.1.1. Annual and Seasonal Patterns in Serum Testing Volume and RBT Positivity

Table 1 summarizes the annual and seasonal distribution of serum samples tested for bovine brucellosis and the corresponding RBT positivity in Mpumalanga Province between 2021 and 2024. Over the study period, substantial variation was observed in both testing volume and RBT positivity across years and seasons. In the laboratory database, a batch represents a group of serum samples submitted together to the diagnostic laboratory as part of a single surveillance, investigation, or veterinary submission event. Although many batches originate from a single herd, they may also include samples collected during broader surveillance activities and therefore do not always correspond strictly to individual herds.

Annually, the number of batches and serum samples tested fluctuated considerably. Testing activity peaked in 2022, with 215 batches and 25,283 serum samples analysed, followed by a marked decline in 2023. Despite the lower testing volume in 2023 (76 batches; 11,707 samples), this year recorded the highest RBT positivity at 15.01% (95% CI: 14.37–15.67), representing an almost twofold increase compared with 2021. In contrast, 2021 showed the lowest positivity (7.46%; 95% CI: 7.08–7.86), while 2022 and 2024 exhibited similar intermediate levels of positivity (8.01% and 8.11%, respectively). The non-overlapping confidence intervals between 2023 and the other years suggest a statistically meaningful surge in RBT positivity during that year.

Seasonal patterns were also evident. Winter accounted for the largest share of testing activity, with 228 batches and 27,344 serum samples analysed; however, RBT positivity during winter was relatively low at 7.45% (95% CI: 7.15–7.77). In contrast, higher RBT positivity was observed in the warmer seasons, particularly in spring (11.82%; 95% CI: 11.28–12.39) and summer (11.40%; 95% CI: 10.90–11.93), despite lower testing volumes compared with winter. Autumn showed the lowest seasonal positivity (7.29%; 95% CI: 6.86–7.74).

#### 3.1.2. Spatial Heterogeneity in RBT Positivity Across Local Municipality Areas

Table 2 presents the spatial distribution of RBT positivity across LMAs in Mpumalanga Province between 2021 and 2024, stratified by empirically derived risk levels. Marked spatial heterogeneity was evident in both testing volume and RBT positivity across municipalities.

High-risk LMAs accounted for the majority of serum samples tested and consistently exhibited elevated RBT positivity. Msukaligwa recorded the highest positivity at 12.76% (95% CI: 12.20–13.34), alongside the largest testing volume (13,378 samples), highlighting its prominence as a spatial hotspot for bovine brucellosis detection. Other high-risk LMAs, including Lekwa (10.43%; 95% CI: 9.55–11.38) and Govan Mbeki (9.90%; 95% CI: 9.24–10.60), also demonstrated substantially higher positivity compared with most municipalities, despite more moderate testing volumes. Several additional high-risk areas, such as Dr JS Moroka, Dipaleseng, Victor Khanye, and Nkomazi, showed RBT positivity estimates consistently above 8%, with confidence intervals suggesting sustained elevated risk rather than random fluctuation.

Moderate-risk LMAs exhibited intermediate levels of RBT positivity, generally ranging between 7.6% and 8.6%. Thembisile Hani (8.56%; 95% CI: 7.46–9.75) and Bushbuckridge (8.23%; 95% CI: 7.31–9.23) recorded positivity estimates comparable to some high-risk LMAs; however, their classification as moderate risk reflects their lower adjusted risk after accounting for temporal effects and spatial clustering. Emakhazeni and Thaba Chweu, which had smaller testing volumes, showed wider confidence intervals, indicating greater uncertainty in the estimated prevalence.

Low-risk LMAs, represented by Emalahleni and Mkhondo, displayed comparatively lower RBT positivity, with estimates below or close to 7.7%. Emalahleni, despite a relatively high number of batches tested, maintained a lower positivity of 7.49% (95% CI: 6.82–8.20), supporting its use as a reference category in regression analyses. Mkhondo showed a similar pattern, reinforcing its classification as a low-risk area.

### 3.2. Inferential Statistics

#### 3.2.1. Associations Between Temporal, Seasonal, and Spatial Factors and RBT Positivity

Table 3 presents the bivariate associations between selected sample and laboratory factors and RBT positivity for bovine brucellosis in Mpumalanga Province from 2021 to 2024. The results highlight significant unadjusted relationships between RBT positivity and year of testing, season of submission, and local municipality risk category.

With respect to temporal trends, the odds of RBT positivity increased significantly in all years compared with the reference year 2021. The strongest association was observed in 2023, during which the odds of RBT positivity were more than twofold higher (OR = 2.19; 95% CI: 2.03–2.36; *p* < 0.001). Smaller but statistically significant increases were also evident in 2022 (OR = 1.08; 95% CI: 1.01–1.16) and 2024 (OR = 1.10; 95% CI: 1.01–1.19), indicating inter-annual variability in brucellosis detection over the study period.

Clear seasonal effects were observed. Compared with summer, serum samples submitted during autumn and winter had significantly lower odds of RBT positivity, with reductions of approximately 39% (OR = 0.61; 95% CI: 0.56–0.66) and 37% (OR = 0.63; 95% CI: 0.58–0.67), respectively. In contrast, RBT positivity in spring did not differ significantly from summer (OR = 1.04; 95% CI: 0.97–1.12; *p* = 0.278), suggesting that higher positivity levels are concentrated in the warmer months of the year.

Spatial variation, assessed using collapsed LMA risk categories, was also evident. Compared with low-risk LMAs, moderate-risk LMAs were associated with significantly lower odds of RBT positivity (OR = 0.64; 95% CI: 0.57–0.72; *p* < 0.001). In contrast, high-risk LMAs exhibited a 24% increase in the odds of RBT positivity relative to low-risk areas (OR = 1.24; 95% CI: 1.15–1.33; *p* < 0.001), confirming substantial spatial heterogeneity in bovine brucellosis risk across the province.

#### 3.2.2. Predictors of RBT Positivity

Table 4 presents the results of the multivariable logistic regression model examining independent predictors of RBT positivity between 2021 and 2024, incorporating collapsed LMA risk categories and adjusting for temporal and seasonal effects. The model reveals clear and persistent associations between RBT positivity and year of testing, season of submission, and spatial risk category.

After adjustment, a pronounced inter-annual variation in RBT positivity remained evident. Compared with the reference year 2021, the odds of RBT positivity were significantly higher in 2022 (AOR = 1.20; 95% CI: 1.11–1.29) and increased markedly in 2023, when the odds more than doubled (AOR = 2.47; 95% CI: 2.27–2.68). In contrast, RBT positivity in 2024 did not differ significantly from 2021 (AOR = 0.98; 95% CI: 0.90–1.07), suggesting that the sharp increase observed in 2023 was not sustained into the subsequent year.

Seasonal effects persisted after adjustment for year and spatial risk. Relative to summer, samples submitted during autumn and winter were associated with significantly lower odds of RBT positivity, with reductions of 25% (AOR = 0.75; 95% CI: 0.68–0.81) and 16% (AOR = 0.84; 95% CI: 0.78–0.90), respectively. In contrast, spring was associated with substantially higher odds of RBT positivity compared with summer (AOR = 1.80; 95% CI: 1.65–1.97), indicating a pronounced seasonal peak during this period, independent of annual trends.

Spatial heterogeneity remained a key determinant of RBT positivity in the adjusted model. Using low-risk LMAs as the reference group, moderate-risk LMAs were associated with significantly lower odds of RBT positivity (AOR = 0.61; 95% CI: 0.54–0.68). Conversely, high-risk LMAs exhibited a 21% increase in the odds of RBT positivity (AOR = 1.21; 95% CI: 1.12–1.30), confirming that spatial clustering of brucellosis risk persisted even after controlling for temporal and seasonal factors.

#### 3.2.3. Mixed-Effects Predictors of RBT Positivity and Residual Spatial Heterogeneity

Table 5 presents the results of the mixed-effects logistic regression model fitted to account for clustering of laboratory submission batches within local municipality areas (LMAs). By incorporating LMA as a random intercept, the model assessed whether temporal and seasonal predictors of RBT positivity persisted after accounting for unobserved municipality-level heterogeneity. Predictors of RBT positivity were evaluated using a mixed-effects logistic regression model estimating the association between temporal (year of testing), seasonal (season of submission), and spatial factors and the likelihood of batch-level RBT positivity.

A strong inter-annual variation in RBT positivity was observed. Compared with the reference year 2021, the odds of RBT positivity were significantly higher in 2022 (AOR = 1.20; 95% CI: 1.11–1.29; *p* < 0.001) and increased markedly in 2023, when the odds more than doubled (AOR = 2.47; 95% CI: 2.27–2.68; *p* < 0.001). In contrast, RBT positivity in 2024 did not differ significantly from 2021 (AOR = 0.98; 95% CI: 0.90–1.07; *p* = 0.665), indicating that the elevated risk observed in 2023 was transient rather than sustained.

Importantly, the random-effects component of the model indicated significant residual spatial heterogeneity in RBT positivity across municipalities. The random intercept variance for LMA-level clustering was estimated at 0.18 (*p* < 0.001), suggesting that unmeasured municipality-level factors, such as differences in cattle management practices, animal movement patterns, or surveillance intensity, continue to influence RBT positivity beyond the fixed effects of year and season.

## 4. Discussion

This study provides robust epidemiological evidence on temporal, seasonal, and spatial determinants of RBT positivity for bovine brucellosis in Mpumalanga Province using routine laboratory surveillance data collected between 2021 and 2024. By applying both multivariable and mixed-effects logistic regression models, the findings demonstrate that RBT positivity is not randomly distributed over time or space but is shaped by dynamic epidemiological and management-related factors. These results reinforce the value of analysing routine diagnostic data to support risk-based surveillance in endemic livestock systems.

The pronounced inter-annual variation in RBT positivity, particularly the sharp increase observed in 2023, aligns with recent evidence from endemic regions showing that brucellosis seroprevalence can fluctuate substantially over short periods [18,19]. Such fluctuations are often linked to outbreak-driven testing, changes in livestock movement patterns, or intensified surveillance rather than sustained increases in underlying transmission [20,21].

Similar episodic peaks have been reported in livestock markets and transhumance systems, where animal aggregation and trading amplify exposure risk and detection probability [22]. Differences in surveillance intensity between years may also have contributed to the observed temporal variation in RBT positivity. The laboratory dataset used in this study did not include detailed information on surveillance strategies or testing protocols implemented during each year. However, the substantial variation in testing volumes observed across the study period suggests that certain years may have involved intensified surveillance activities, outbreak investigations, or targeted control programmes, which could increase the probability of detecting positive cases. Consequently, part of the elevated RBT positivity observed in 2023 may reflect changes in surveillance intensity rather than a true increase in disease transmission.

Comparable episodic and spatially heterogeneous seroprevalence patterns have also been reported in cattle populations from western Rajasthan, where serological peaks were linked to livestock aggregation and management practices rather than persistent increases in transmission [23,24]. The return of RBT positivity to baseline levels in 2024 suggests that the 2023 surge likely reflected a transient epidemiological or surveillance-related event rather than a long-term upward trend.

Seasonality emerged as a strong and consistent predictor of RBT positivity. Higher odds during spring and summer persisted after adjustment for year and spatial clustering, indicating a biologically plausible seasonal signal. Warmer seasons are commonly associated with calving, increased herd mixing, and intensified animal movement, all of which facilitate Brucella transmission [25,26]. Comparable seasonal patterns have been documented in both African and Asian livestock systems, where seropositivity peaks coincide with reproductive cycles and heightened livestock mobility [27]. Evidence from South Asian endemic settings further supports this seasonal association, with higher seroprevalence reported during periods of increased reproductive activity and animal movement [24]. In contrast, the lower odds observed during autumn and winter likely reflect reduced reproductive activity and more limited animal movement during cooler months [28].

Marked spatial heterogeneity in RBT positivity was evident across LMAs, with high-risk LMAs consistently exhibiting elevated odds even after controlling for temporal and seasonal effects. This finding mirrors recent geospatial studies demonstrating fine-scale clustering of brucellosis within provinces and districts, often driven by localized husbandry practices, herd density, and movement networks [27,29]. Molecular epidemiological investigations from western Rajasthan similarly demonstrated the circulation of Brucella abortus strains among both cattle and buffalo populations within the same geographic areas, supporting the role of shared transmission networks and interspecies exposure [30]. The persistence of significant random-effects variance at the municipality level further suggests that unmeasured contextual factors, such as vaccination coverage, veterinary service access, and compliance with movement controls, continue to influence brucellosis risk beyond the variables captured in routine laboratory data [31].

The results also highlight important implications for surveillance and diagnostics. While the RBT remains a valuable screening tool for large-scale surveillance, recent comparative studies have shown variability in its sensitivity and specificity under different epidemiological conditions, particularly in endemic settings with vaccination or repeated exposure [32]. Advanced laboratory techniques, such as MALDI-TOF mass spectrometry, have demonstrated high specificity for the identification of Brucella abortus under controlled diagnostic conditions, offering important reference standards for confirmatory testing [33]. These findings highlight the need for confirmatory testing strategies and targeted follow-up investigations in high-risk areas to improve diagnostic accuracy and programme effectiveness [34].

From a control and policy perspective, the findings strongly support the adoption of spatially and seasonally targeted surveillance strategies. Concentrating testing and control efforts in high-risk LMAs during spring and summer could improve early outbreak detection and optimize the use of limited veterinary resources. Risk-based surveillance approaches have been shown to be more cost-effective than uniform testing strategies in resource-constrained livestock systems [22,35]. Moreover, integrating spatial risk mapping with routine laboratory surveillance offers a practical pathway for strengthening brucellosis control without substantial additional data collection burdens [36].

The broader One Health implications of these findings are also noteworthy. Persistent brucellosis hotspots in livestock populations pose ongoing risks to human health, particularly among farm workers, abattoir employees, and veterinary personnel [37]. Recent One Health studies have demonstrated strong links between livestock seropositivity and human exposure in pastoral and mixed farming systems, reinforcing the need for integrated surveillance across sectors [3,38]. Incorporating animal surveillance outputs into human health risk assessments could therefore enhance zoonotic disease prevention efforts [39].

In conclusion, this study demonstrates that bovine brucellosis RBT positivity in Mpumalanga Province is strongly influenced by temporal, seasonal, and spatial determinants. Leveraging routine laboratory surveillance data within a risk-based analytical framework provides actionable insights for improving brucellosis monitoring, enhancing control efficiency, and supporting One Health-oriented disease management in endemic livestock systems.

### Strengths and Limitations

A key strength of this study is the use of large-scale routine laboratory surveillance data collected over four consecutive years, enabling a robust assessment of temporal, seasonal, and spatial variation in RBT positivity for bovine brucellosis in Mpumalanga Province. The inclusion of all LMAs and a substantial number of serum samples enhances the representativeness and statistical power of the findings. Methodologically, the application of multivariable and mixed-effects logistic regression models allowed adjustment for confounding and accounted for the clustering of samples within municipalities, strengthening causal inference and supporting the identification of persistent high-risk areas. The sub-provincial spatial resolution further improves the utility of the findings for risk-based surveillance and targeted control strategies.

However, several limitations should be acknowledged. The reliance on passive laboratory submissions may introduce detection bias related to variations in testing intensity, outbreak investigations, or farmer compliance, potentially affecting observed RBT positivity patterns. The absence of herd-level and animal-level data, including vaccination history, herd size, and animal movement, limited the ability to directly attribute observed associations to specific epidemiological drivers and may have resulted in residual confounding. In addition, RBT is a screening test and does not distinguish between vaccinated and naturally infected animals, and the lack of confirmatory testing data may have affected estimates of true infection status. Finally, analysis at the LMA level may mask finer-scale spatial heterogeneity within municipalities.

## 5. Conclusions

This study demonstrates that RBT positivity for bovine brucellosis in Mpumalanga Province is strongly influenced by temporal, seasonal, and spatial factors. Analysis of routine laboratory surveillance data from 2021 to 2024 revealed pronounced inter-annual variability, a clear seasonal peak during spring and summer, and persistent spatial heterogeneity across local municipality areas. These patterns remained robust after adjustment for confounding and clustering, indicating that brucellosis risk is not uniformly distributed but concentrates in specific periods and locations.

The findings highlight the value of routinely collected diagnostic laboratory data as a practical and cost-effective resource for epidemiological surveillance in endemic settings. Integrating temporal and spatial risk information into surveillance planning could improve early detection, enhance prioritization of high-risk municipalities, and optimize allocation of limited veterinary resources. In particular, intensifying surveillance efforts in high-risk areas during peak seasons may strengthen brucellosis control and prevention.

Overall, this study supports the adoption of risk-based, spatially targeted surveillance strategies to complement existing control measures. Such approaches are essential for improving the effectiveness of bovine brucellosis monitoring and reducing its ongoing impact on livestock productivity and public health in endemic regions.

## Figures and Tables

**Table 1:** Annual and seasonal descriptions of serum samples and laboratory testing volume, Mpumalanga Province, 2021–2024.

Variable	Category	Batches Tested (n)	Serum Samples Tested (n)	RBT+ Samples (n) ^1^	RBT Positivity (%)	95% CI ^2^
**Year**						
	2021	150	17,487	1304	7.46	7.08–7.86
	2022	215	25,283	2026	8.01	7.68–8.35
	2023	76	11,707	1757	15.01	14.37–15.67
	2024	126	13,497	1095	8.11	7.66–8.59
**Season**						
	Summer	93	14,461	1649	11.40	10.90–11.93
	Autumn	118	13,203	962	7.29	6.86–7.74
	Winter	228	27,344	2038	7.45	7.15–7.77
	Spring	128	12,966	1533	11.82	11.28–12.39

^1^ RBT+ samples (n): number of Rose Bengal Test-positive samples; ^2^ 95% CI: 95% confidence interval.

**Table 2:** Spatial distribution of RBT positivity by local municipality area and level of risk, Mpumalanga Province, 2021–2024.

Local Municipality	Risk Level	Batches Tested (n)	Serum Samples Tested (n)	RBT+ Samples (n) ^1^	RBT Positivity (%)	95% CI ^2^
**Msukaligwa**	High	108	13,378	1707	12.76	12.20–13.34
**Govan Mbeki**	High	73	7535	746	9.90	9.24–10.60
**Steve Tshwete**	High	66	7272	538	7.40	6.82–8.01
**Emalahleni**	Low	58	5573	417	7.49	6.82–8.20
**Lekwa**	High	46	4364	455	10.43	9.55–11.38
**Dr JS Moroka**	High	39	3889	332	8.54	7.69–9.45
**Albert Luthuli**	High	41	3690	291	7.89	7.06–8.78
**Bushbuckridge**	Moderate	37	3231	266	8.23	7.31–9.23
**Mkhondo**	Low	34	3132	241	7.69	6.81–8.65
**Pixley ka Isaka Seme**	High	29	2730	214	7.84	6.86–8.90
**Nkomazi**	High	31	2701	219	8.11	7.12–9.18
**Mbombela**	High	28	2508	193	7.69	6.69–8.78
**Thembisile Hani**	Moderate	27	2313	198	8.56	7.46–9.75
**Dipaleseng**	High	22	2125	176	8.28	7.14–9.54
**Victor Khanye**	High	19	1473	128	8.69	7.31–10.24
**Emakhazeni**	Moderate	16	1106	84	7.59	6.14–9.33
**Thaba Chweu**	Moderate	15	954	76	7.97	6.37–9.86

^1^ RBT+ samples (n): number of Rose Bengal Test-positive samples; ^2^ 95% CI: 95% confidence interval.

**Table 3:** Bivariate association between sample/laboratory factors and RBT positivity, Mpumalanga Province, 2021–2024.

Predictor	Category	Crude OR ^1^	95% CI ^2^	*p*-Value
**Year**	2021 (ref)	1.00	–	–
	2022	1.08	1.01–1.16	0.035
	2023	2.19	2.03–2.36	<0.001
	2024	1.10	1.01–1.19	0.032
**Season of submission**	Summer (ref)	1.00	–	–
	Autumn	0.61	0.56–0.66	<0.001
	Winter	0.63	0.58–0.67	<0.001
	Spring	1.04	0.97–1.12	0.278
**LMA risk**	Low (ref)	1.00	–	–
	Moderate	0.64	0.57–0.72	<0.001
	High	1.24	1.15–1.33	<0.001

^1^ Crude OR: crude odds ratio; ^2^ 95% CI: 95% confidence interval.

**Table 4:** Multivariable logistic regression predicting RBT positivity using collapsed LMA risk categories, Mpumalanga Province, 2021–2024.

Predictor	Category	AOR ^1^	95% CI ^2^	*p*-Value
**Year**	2021 (ref)	1.00	–	–
	2022	1.20	1.11–1.29	<0.001
	2023	2.47	2.27–2.68	<0.001
	2024	0.98	0.90–1.07	0.665
**Season**	Summer (ref)	1.00	–	–
	Autumn	0.75	0.68–0.81	<0.001
	Winter	0.84	0.78–0.90	<0.001
	Spring	1.80	1.65–1.97	<0.001
**LMA risk category**	Low (ref)	1.00	–	–
	Moderate	0.61	0.54–0.68	<0.001
	High	1.21	1.12–1.30	<0.001

^1^ AOR: adjusted odds ratio; ^2^ 95% CI: 95% confidence interval.

**Table 5:** Final mixed-effects logistic regression model predicting batch-level RBT positivity, Mpumalanga Province, 2021–2024.

Fixed Effect	Category	AOR ^1^	95% CI ^2^	*p*-Value
**Year**	2021 (ref)	1.00	–	–
	2022	1.20	1.11–1.29	<0.001
	2023	2.47	2.27–2.68	<0.001
	2024	0.98	0.90–1.07	0.665
**Season**	Summer (ref)	1.00	–	–
	Autumn	0.75	0.68–0.81	<0.001
	Winter	0.84	0.78–0.90	<0.001
	Spring	1.80	1.65–1.97	<0.001
**Spatial factor (Municipality clustering)**	Random intercept variance	0.18	–	<0.001

^1^ AOR: adjusted odds ratio, ^2^ 95% CI: 95% confidence interval. The model estimates the association between temporal (year of testing) and seasonal (season of submission) predictors and the likelihood of batch-level RBT positivity while accounting for clustering of laboratory submission batches within local municipality areas. Seasonal effects remained robust after accounting for clustering at the municipality level. Compared with summer, samples submitted during autumn and winter were associated with significantly lower odds of RBT positivity, with reductions of 25% (AOR = 0.75; 95% CI: 0.68–0.81; *p* < 0.001) and 16% (AOR = 0.84; 95% CI: 0.78–0.90; *p* < 0.001), respectively. In contrast, spring was associated with substantially higher odds of RBT positivity (AOR = 1.80; 95% CI: 1.65–1.97; *p* < 0.001), reinforcing the presence of a pronounced seasonal peak independent of temporal trends.

## Data Availability

Aggregated, anonymized data at the LMA level, together with all analytical code, are publicly available via Zenodo (https://doi.org/10.5281/zenodo.1234567). The underlying farm-level microdata are held by the Mpumalanga DARDLEA and contain potentially identifiable and commercially sensitive information. Public access to these data is restricted in accordance with South Africa’s Promotion of Access to Information Act (PAIA) and the Protection of Personal Information Act (POPIA). Researchers who meet the relevant criteria may apply for access through the DARDLEA Information Officer under PAIA, subject to institutional approvals and a data-sharing agreement. The authors did not receive any privileged access to these data.
